# Exercise referral schemes increase Patients’ cardiorespiratory Endurance: A systematic review and Meta-Analysis

**DOI:** 10.1016/j.pmedr.2024.102844

**Published:** 2024-08-03

**Authors:** Sophie J.L. Inkpen, Haoxuan Liu, Sophie Rayner, Ellie Shields, Judith Godin, Myles W. O’Brien

**Affiliations:** a Division of Kinesiology, Dalhousie University, Halifax, Nova Scotia B3H 4R2, Canada; b Faculty of Kinesiology, Sport, and Recreation, University of Alberta, Edmonton, Alberta T6G 2H9, Canada; c Medical Sciences, Dalhousie University, Halifax, Nova Scotia B3H 4R2, Canada; d Geriatric Medicine Research, Nova Scotia Health, Halifax, Nova Scotia B3H 4R2, Canada; e Department of Medicine, Université de Sherbrooke, Sherbrooke, Quebec, Canada; f Centre de Formation Médicale du Nouveau-Brunswick, Université de Sherbrooke, Moncton, New Brunswick, Canada

**Keywords:** Cardiorespiratory fitness, Exercise prescription, Primary health care, physical activity counselling, Exercise is Medicine

## Abstract

**Introduction:**

The efficacy of exercise referral schemes (ERS) involving primary care providers to an exercise specialist on patients’ physical activity is uncertain and primarily based on self-report outcomes. Cardiorespiratory endurance carries clinically relevant information and is an objective outcome measure that has been used to evaluate ERS, but this literature has not been amalgamated. We determined the effectiveness of ERS involving qualified exercise professionals (QEPs) on patients’ cardiorespiratory endurance.

**Methods:**

A systematic review with between-group and within-group *meta*-analyses was performed to examine the effects of ERS on cardiorespiratory endurance. We searched Scopus, EMBASE, MEDLINE, CINAHL, and Academic Search Premier databases from their inception to February 2023 to find ERS interventions (randomized/non-randomized, controlled/non-controlled). To be included, studies required an adult patient referral from a primary care provider to a QEP.

**Results:**

Twenty-nine articles comprising 6326 (3684 females) unique patients were included. Patients were primarily older (62 ± 9 years; range: 48–82) and overweight (body mass index: 28.9 ± 7.5 kg/m^2^; range: 22.5–37.1). Improvements in patients’ cardiorespiratory endurance were observed in 20 of the 29 studies. Among controlled studies (*n* = 14), the *meta*-analysis exhibited a favorable effect on cardiorespiratory endurance between the intervention and the comparator groups (Hedge’s g: 0.31, 95 % CI: 0.09 to 0.52). The ERS interventions also improved cardiorespiratory endurance when comparing pre- and post-intervention effects (all studies, Cohen’s *d*: 0.57, 95 % CI: 0.45 to 0.69).

**Conclusion:**

ERS that incorporate a QEP lead to improvements in patients’ cardiorespiratory endurance, providing support for the creation of these programs to help patients lead healthier lifestyles.

## 1 Introduction

The health benefits of leading a physically active lifestyle and maintaining a high level of cardiorespiratory endurance are well-established (Blair et al., 2001, Ross et al., 2016). Cardiorespiratory endurance or fitness represents the greatest amount of oxygen that can be delivered and extracted from inspired air while performing dynamic exercise involving a large percentage of total body muscle mass (Ross et al., 2016). With age and physical inactivity, cardiorespiratory endurance decreases (Woo et al., 2006) which represents an increased likelihood of developing cardiovascular diseases and experiencing a cardiovascular event. Conversely, increasing cardiorespiratory endurance levels translates to health improvements. Specifically, a 1 metabolic equivalent of task increase in cardiorespiratory endurance may confer a 12–35 % improvement in survival risk and a 15 % risk reduction of all-cause mortality (Ross et al., 2016, Mora et al., 2003, Nes et al., 2014). It has been proposed that cardiorespiratory endurance should be considered a vital sign in primary care as it may be a stronger predictor of mortality than smoking, hypertension, high cholesterol, and type 2 diabetes mellitus (Ross et al., 2016). Moreover, cardiorespiratory endurance is directly associated with functional fitness in older adults. It has been indicated that cardiorespiratory endurance below approximately 20 ml/kg/min is the critical threshold at which the probability of maintaining independent living decreases significantly (Posner et al., 1995, Cress and Meyer, 2003, Morey et al., 1998). Accordingly, strategies that target improvements in patients’ cardiorespiratory endurance are imperative for addressing the Western worlds’ high prevalence of chronic disease and multimorbidity (Chowdhury et al., 2023).

Exercise referral schemes (ERS) comprise a referral from primary care providers to exercise professionals for additional patient physical activity counselling, exercise prescription, and/or exercise programming (Pavey et al., 2011). Most of the studies investigating the effectiveness of ERS have implemented self-report measures of physical activity (O’Brien et al., 2021), which may be subject to high degrees of error and lack the precision to detect changes in habitual activity. Conversely, cardiorespiratory endurance is an established measure of physical health that is more stable than habitual activity but is often not considered in reviews of ERS effectiveness. As such, understanding whether ERS improve patients’ cardiorespiratory endurance will provide important insight into potential healthcare interventions to address our inactive population.

Surveys among healthcare providers have demonstrated a strong desire to refer patients to qualified exercise professionals (QEPs) for affordable activity advice and to supervised community exercise programs (Pellerine et al., 2022). A nuance is the requirement for the exercise professionals to be *qualified*. QEPs; such as certified exercise (or clinical) physiologists, registered kinesiologists, and physiotherapists for example, have the background training, knowledge, and scope of practice to effectively provide physical activity counselling, prescribe exercise, and deliver exercise programming for generally healthy individuals and those living with chronic disease (O’Brien et al., 2021, Thornton et al., 2016). A scoping review by our group mapped out that ERS involving qualified exercise professionals (QEPs) generally observe increases in cardiorespiratory endurance (O’Brien et al., 2021), but the type of review did not permit the ability to answer a specific hypothesis-driven research question, examine the quality of evidence on the topic, or quantify the magnitude of the potential change via *meta*-analyses.

The purpose of this study was to systematically review and perform a *meta*-analysis regarding the effectiveness of ERS linking primary care providers and QEPs. It was hypothesized that these intervention schemes would improve patients’ cardiorespiratory endurance level. Exploratory sub-group analyses of different scheme characteristics, study quality, and measurements of cardiorespiratory endurance were conducted.

## 2 Methods

### 2.1 Search strategy

The search strategy and systematic review procedures were registered in Prospero (ID# CRD42023396981) prior to conducting the study. The study is based on publically available data and did not require review from a Research Ethics Board. The PICO (i.e., Population [adults], Intervention [ERS], Comparison [within or between groups], Outcome [cardiorespiratory endurance]) approach was used to develop the search strategy. Literature searches were conducted using Scopus, EMBASE, MEDLINE, CINAHL, and Academic Search Premier databases up to February 5th, 2023. Our search strategy framework is presented in Supplemental Digital Content Table S1. The focus of this review was on patients’ cardiorespiratory endurance and the impact of including studies examining *physical activity* has been reviewed extensively elsewhere (Pellerine et al., 2022, Chowdhury et al., 2023) and is beyond the scope of this review. Other reviews of ERS (Pellerine et al., 2022, Thornton et al., 2016, Campbell et al., 2015, Pavey et al., 2011) and articles citing these reviews were searched for relevant articles in April 2023. This review followed the Preferred Reporting for Items for Systematic Reviews and Meta-Analysis (PRISMA) 2020 statement (Page et al., 2020). Article citations were downloaded to an online research management system (Mendeley, Elsevier, Amsterdam, Netherlands) and duplicates were removed. Remaining references were exported to systematic review software for screening (Covidence, Melbourne, Australia).

### 2.2 Study inclusion and exclusion criteria

Studies not published in a peer reviewed journal, or published as an editorial, opinion, review, or conference abstract were excluded. Grey literature was excluded. No language or timeline restrictions were implemented into the search strategy. All studies included human participants. To answer our research question, most study participants must be referred from primary care and the study had to include a measure of cardiorespiratory endurance. The type of referral scheme intervention was expected to be variable. Only interventional designs were included, specifically randomized and non-randomized (pre-post studies, with or without control groups).

**Fig. 1:**
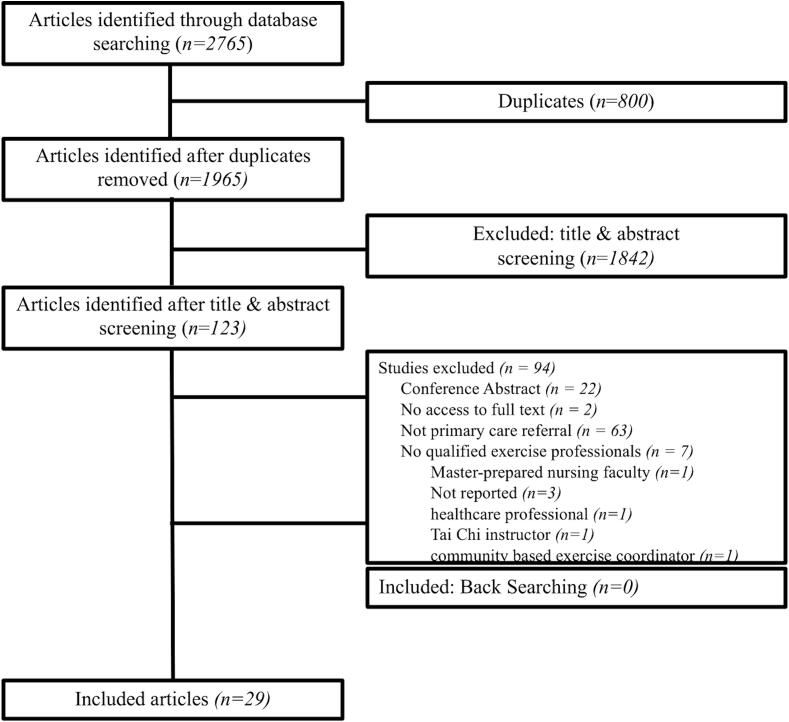
Flow-chart indicating the number of articles included or excluded at each part of the screening process and breakdown of articles.

Studies were included if patients were referred from health care (e.g., primary care provider, primary care clinic, etc.) to a QEP for physical activity counselling (e.g., behavioral counselling), exercise programming, and/or supervised exercise. Individuals with or without a medical diagnosis were included. No time limit was placed on the duration of the intervention or follow-up measurement period. To be considered a QEP, a recognized certification with the training, scope of practice, and liability insurance to cover fitness assessment and exercise prescription for generally healthy and those living with chronic disease or a minimum level of educational training (such as a kinesiology or exercise science degree) was required. Studies in which the intervention was delivered by a research assistant or other healthcare professional (e.g., nurse, dietitian, etc.) without a description of their exercise-related qualifications were excluded (Fig. 1).

### 2.3 Study screening process and data extraction

The titles and abstracts of citations were screened independently by two reviewers who identified potential articles for inclusion. The full text of apparently relevant articles was obtained and screened by the same reviewers. In cases of disagreement, a senior reviewer was consulted and a decision was made by consensus. There were 129 conflicts during titles/abstract screening and 14 conflicts during full-text review. The reference list of included articles was back searched for other potentially relevant articles.

In addition to cardiorespiratory endurance, we extracted the measurement tools, primary care provider and exercise professional information, country of study, patient characteristics (age, sex, disease status, etc.), and intervention characteristics.

### 2.4 Study quality assessment

The National Institutes of Health quality assessment tools for controlled intervention studies or pre-post studies with no control group were used to assess the study quality/bias depending on the study design (i.e., whether a control group was included). The specific tool checklist and information on how to score each tool is presented on the National Institutes of Health website (National Heart Lung and Blood Insitute, 2021). A higher value indicates a higher study quality (Yes = 1; No, cannot determine, or not applicable = 0). The specific tool checklist and information on how to score each tool is presented on the National Institutes of Health website (National Heart Lung and Blood Insitute, 2021).

Similar to the article screening process, quality assessment for each article was independently completed by two reviewers. A third reviewer was consulted to make a final decision in each instance of disagreement between reviewers.

### 2.5 Meta-Analyses

The *meta*-analysis and subgroup analyses were performed using the R software 4.2.3 2023 with the packages *met* (Balduzzi et al., 2019) and *metafor* (Viechtbauer, 2010). For studies that included a control group, the between-group effect size was calculated as the standardized mean difference (SMD_between_, Hedge’s g) with 95 % confidence intervals (CIs) using a random effects model. One study was excluded from the *meta*-analysis due to the unavailability of data for the control group (Lamb et al., 2018). The SMD was calculated using the differences of the change (i.e., Post – Pre) in cardiorespiratory endurance between the intervention and the control groups. If the standard deviations of the change were not available, a correlation coefficient of 0.71 between pre- and post-intervention/control values obtained from an included study (Mustian et al., 2009) was used to calculate the missing standard deviations using the formula provided in the Section 16.1.3.2 of the Cochrane Handbook. In studies with multiple intervention arms, the data were combined using the formulas provided in Section 7.7.3.8 of the Cochrane Handbook (Table 7.7.a) into a single group sample size, mean, and standard deviation.

Regardless of the inclusion of a control group, the within-group effect size (Cohen’s *d*) was calculated as the standardized mean difference (SMD_within_) by taking the mean difference between pre- and post-intervention and dividing it by the standard deviation of the mean difference. However, if a study’s control group also received alternative ERS interventions (e.g., counselling), the data from the intervention and control groups were combined (Section 7.7.3.8 of the Cochrane Handbook). As such, the single-group pre-post design *meta*-analysis of the ERS interventions was conducted to reflect a specific treatment effect. If the mean difference was not reported, the difference of mean values between pre- and post-intervention was calculated. If the standard deviations of the mean difference were not available, the above-mentioned approach (Section 16.1.3.2 of the Cochrane Handbook) was utilized to estimate the values. The standard error of the SMD_within_ was calculated using the formula $\mathrm{SE}=\sqrt{\frac{2(1-r)}{n}+\frac{{\mathrm{SMD}}^{2}}{2n}}$, where *r* represented a correlation coefficient of 0.71, *SMD* was the calculated within-group effect size, and *n* referred to the sample size.

The statistical heterogeneity across different studies in the *meta*-analysis was assessed by the I^2^ statistics, where I^2^ ≤ 25 % indicated low heterogeneity, 25–75 % indicated moderate heterogeneity, and ≥ 75 % indicated high heterogeneity. Publication bias was assessed by visual inspection of funnel plots, Galbraith plots (Supplemental Digital Content Figs. S1–S3), and by Egger’s regression test (Egger et al., 1997). Subgroup analyses of the SMD_between_ were performed to examine whether the changes in cardiorespiratory endurance are mediated by the categories of the comparator group [usual care (or no intervention) vs. alternative ERS (e.g., counselling or alternative exercise intervention)], types of ERS (exercise only vs. exercise & counselling vs. counselling only), duration of the intervention (<12-week vs. ≥ 12-week), study quality (NIH quality score above vs below the median value), or cardiorespiratory endurance test (6-min walk test vs. step test vs. maximal cycling test vs. submaximal cycling test vs. maximal treadmill test). Subgroup analyses of the SMD_within_ were performed for the duration of the intervention and cardiorespiratory endurance test. A random-effects model was used to combine studies within each subgroup. Tau-squared (τ^2^) was computed within subgroups and then pooled across subgroups to explore statistical heterogeneity. The subgroup analyses results were presented for each level of factor as pooled SMD_between/within_ with 95 % CI. Statistical significance was defined as *p* < 0.05.

## 3 Results

### 3.1 Study screening

Our search yielded 2765 articles, across MEDLINE (n = 907), EMBASE (n = 858), Scopus (n = 495), Academic Search Premier (n = 258), and CINAHL (n = 257). After duplicates (n = 800) were removed, 1965 articles were screened. As presented in Fig. 1, 29 articles met our inclusion criteria after full-text screening (Lamb et al., 2018, Armit et al., 2009, Perez-Sousa et al., 2020, Sørensen et al., 2008, Ås et al., 2014, Lerdal et al., 2013, Burtscher et al., 2009, Isaacs et al., 2007, Leach et al., 2018, Hameed et al., 2021, Mustian et al., 2009, Marsden et al., 2016, Fortier et al., 2011, Lou et al., 2023, McGrillen and McCorry, 2014, López-Román et al., 2020, Temel et al., 2009, Harman et al., 2021, Jeejeebhoy et al., 2017, Brovold et al., 2012, Knight and Petrella, 2014, Knight et al., 2014, Batsis et al., 2021, Batsis et al., 2021, Tremblay et al., 2020, Meyer et al., 2015, voorn et al., 2021, Delbaere et al., 2006, Meseguer Zafra et al., 2018).

### 3.2 Participant and QEP characteristics

The total number of participants was *n* = 6326 (3684 females), with a range of *n* = 11–2962 (females range: *n* = 0–2399), as presented in Table 1. Participants were 62 ± 9 years (range: 47–82 years) with a body mass index of 28.9 ± 7.5 kg/m^2^ (22.5–37.1 kg/m^2^).

**Table 1:** Study and participant characteristics for controlled studies.

Study	Location	Sample Size (Female)	Age (SD)	QEP involved	Supervision
Sørensen et al., 2008	Denmark	22 (NR)	53.4 (49.8–57.1)	PT	yes
Armit et al., 2009	Australia	136 (NR)	58 (50–70)	Exercise scientist	yes
Romé 2014	Sweden	178 (143)	HD: 57 (12)LD: 56 (12)	PT	I1: no I2: yes
Burtscher et al., 2009	Austria	36 (10)	57.5 (6.9)	Health promotion and exercise physiology specialists	yes
Isaacs et al., 2007	England	520 (NR)	57.04 (8.73)	Exercise instructors	yes
Mustian et al., 2009	US	38 (27)	I: 56.5(13.7)C: 63.3 (9.4)	Certified exercise scientist	no
Marsden et al., 2016	Australia	20 (NR)	I: 54.4 (22.2)C: 62.0 (16.8)	A neurological PT and exercise scientist	no
Fortier et al., 2011	Canada	98 (NR)	Brief: 47.5 (11)Intensive: 47.2 (11.3)	Certified fitness consultant	yes
Lamb et al., 2018	UK	415 (163)	C: 78.4 (7.6) I: 76.9 (7.9)	PT and exercise assistant	yes
Brovold et al., 2012	Norway	77 (NR)	I: 79(6.5)C: 79(6.9)	PT	yes
Meyer et al., 2015	Germany	21 (13)	T: 54(11)C: 59(9)	Educated trainers in exercise therapy and physiotherapy, and a physician	yes
Delbaere et al., 2006	Belgium	54 (NR)	C: 82.18(6.50) I: 82.29 (4.97)	PT	yes
Perez-Sousa et al., 2020	Spain	377 (344)	C: 70.2 (8.0)I: 68.4 (7.0)	Sport science graduates	yes
Knight et al., 2014	Canada	60 (36)	CT: 62(5) SB: 63(4) EX: 63(5)CC: 62(4)	Certified Exercise Physiologist	no
Hameed et al., 2021	US	53 (NR)	VPT: 60 (14) HPT: 57 (14) IE: 59 (20)None: 58 (18)	PT	yes

SD: standard deviation; PT: physiotherapist; NR: not reported; HD: high dose; LD: low dose; EG: exercise group; CG: control group; I: intervention; C: control; NR: not reported; SB: sedentary behaviour; CT: control group; CC: comprehensive counselling; EX: exercise; VPT: virtual physical therapy; HPT: home physical therapy; IE: independent exercise program.

The QEPs included a variety of professions with the main specialty being physiotherapy (*n* = 15/29; see Table 1). Other QEP included Exercise Scientists (7/29), Physiologists (5/29), and exercise instructors (1/29).

### 3.3 Characteristics of the ERS and cardiorespiratory endurance measurement

Most ERS included moderate aerobic (*n* = 10/29), combined aerobic and strength training (*n* = 16/29), strength training only (*n* = 1/29), or non-identified exercise (*n* = 2/29; Table 1). Interventions included 1–7 sessions a week ranging from 10 to 105 min in length. The average session length was 51.6 ± 20.5 min. Most studies supervised their exercise sessions (*n* = 26/29). In the case of the unsupervised interventions, participants were given a written explanation of training. A post-intervention follow-up period was included in 14 of the 29 studies, re-assessing fitness outcomes in the months after the intervention concluded. The follow up length varied from 2 weeks to one year (Supplemental Digital Content Table S2). Other studies assessed fitness outcomes only immediately at the end of the intervention period. Interventions lasted 1–12 months (average 5.5 ± 3.9 months). Most studies used the 6-minute walk test (6MWT) (n = 11/29) to measure cardiorespiratory endurance. Other studies used the 2-km walk test (n = 2), 1-mile walk test (n = 1), 400-m walk test (n = 1), submaximal step test (n = 4), submaximal cycle ergometer test (n = 2), submaximal treadmill test (n = 3), maximal cycle ergometer test (n = 3), and maximal treadmill test (n = 2) (Supplemental Digital Content Table S2). Cardiorespiratory endurance metrics included maximal oxygen consumption (n = 3), estimated or predicted maximal oxygen consumption (n = 10), maximum cycling time (n = 1), maximum walking distance (n = 11), peak heart rate (n = 1), peak aerobic power (n = 1), total walking time (n = 1), and total steps completed (n = 1). An improvement in cardiorespiratory endurance was observed in *n* = 20/29 studies.

### 3.4 Study quality

For the controlled intervention studies (n = 15/29), the average quality score was 9.3/14 (Supplemental Digital Content Table S3). For studies without a control group (n = 14/29) the average score was 7.2/12 (Supplemental Digital Content Table S4). As presented in Table 2, all controlled studies indicated similar baseline characteristics (n = 14/14), consistent underlying treatments, and reliable assessment of outcomes. Ten of the 14 controlled trials randomized participants. Dropout was less than 20 % in 7 of the 15 controlled studies and 8 of 14 studies without a control group (Supplemental Digital Content Table S3 & Table S4). Providers were blinded to participants outcomes in n = 2/14 controlled studies, but in 0/14 studies without a control group (Supplemental Digital Content Table S3 & Table S4).

**Table 2:** Study and Participant Characteristics for uncontrolled studies.

Study	Location	Sample Size (Female)	Age (SD)	QEP involved	Supervision
Lerdal et al., 2013	Norway	57 (46)	47.6 (11.7)	PT	yes
Leach et al., 2018	US	169 (91)	63.7 (12.2)	PT	yes
Lou et al., 2023	Australia	21 (3)	65.5 (NR)	Certified Exercise Physiologist	yes
McGrillen and McCorry, 2014	UK	14 (2)	61 (NR)	PT	yes
López-Román et al., 2020	Spain	2962 (2399)	53.5 (NR)	Graduate in Sciences of Physical Activities and Sports	yes
Temel et al., 2009	US	11(NR)	68 (48–81)	PT	yes
Harman et al., 2021	US	210 (NR)	LOB: 61.9 (13.6) AOC: 66.5 (12.7)	Exercise physiologist and certified Clinical Cancer Exercise Specialists	yes
Jeejeebhoy et al., 2017	Canada	253 (1 3 1)	59.1 (9.7)	Clinical Kinesiologist	yes
Knight et al., 2014	Canada	20 (12)	CT=62(5) SB=63(4) EX=63(5) CC=62(4)	Certified Exercise Physiologist	CT: no, CC: yes, SB: yes, EX: no
Batsis et al., 2021	US	28 (23)	72.9 (5.3)	PT	yes
Tremblay et al., 2020	Canada	192 (95)	59.84 (0.66)	Clinical Exercise Specialist	yes
Batsis et al., 2021	US	44 (NR)	73.2 (3.9)	PT	yes
Voorn et al., 2021	Netherlands	26 (NR)	58 (20–76)	PT, Certified Exercise Physiologists	yes
Zafra 2018	Spain	214 (1 3 4)	52.0 (8.5)	Certified physical activity and sports professionals	yes

SD: standard deviation; QEP: qualified exercise professionals; PT: physiotherapist; NR: not reported; LOB: undergone a lobectomy; AOC: all other cancer patients; SB: sedentary behaviour; CT: control group; CC: comprehensive counselling; EX: exercise.

### 3.5 Meta-Analysis of controlled studies

Fourteen studies, comprising 1261 participants (841 intervention and 420 control), were included for *meta*-analysis to compare cardiorespiratory endurance outcomes between the intervention and the comparator groups.

**Fig. 2:**
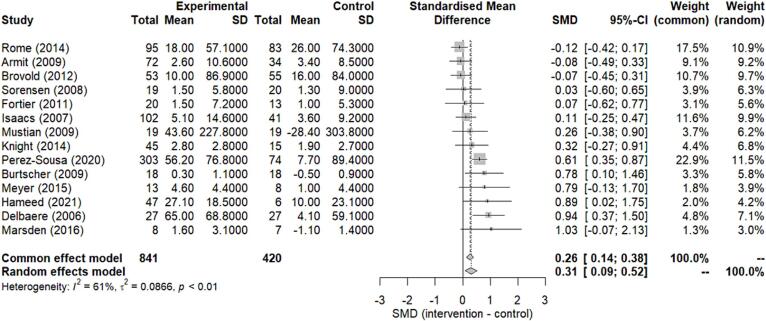
Standardized mean difference (SMD) of the change in cardiorespiratory endurance between the intervention and control groups. Squares represent the SMD for each study. Diamonds represent the pooled SMD across all studies. SD: standard deviation; CI: confidence interval.

The changes in cardiorespiratory endurance outcomes along with the 95 % CIs between the intervention and control groups are summarized in a forest plot (Fig. 2). There was a significant improvement in cardiorespiratory endurance in the ERS intervention group compared to the comparator group [SMD_between_ (95 % CI): 0.31 (0.09 to 0.52); Fig. 2] according to the random-effects model. However, there was evidence of moderate heterogeneity across studies (I^2^ = 61 %, *p* < 0.01; Fig. 2).

Subgroup analyses for the overall effect of ERS according to categories of the comparator group, types of ERS, duration of the intervention, study quality, and cardiorespiratory endurance test are summarized in Table 3. A significant increase in cardiorespiratory endurance was observed in studies including a comparator group that received usual care [SMD_between_ (95 % CI): 0.40 (0.13 to 0.68)], but not in those used an alternative ERS group as a comparator group [SMD_between_ (95 % CI): 0.15 (−0.20 to 0.50)]. When comparing the effects of different types of ERS utilized in the intervention group, only studies that included exercise training as the sole ERS intervention observed a positive effect [SMD_between_ (95 % CI): 0.44 (0.15 to 0.73); Table 3]. In addition, cardiorespiratory endurance was improved when the duration of the intervention was equal to or greater than 12-week [SMD_between_ (95 % CI): 0.31 (0.06 to 0.56)], but not in those duration shorter than 12-week [SMD_between_ (95 % CI): 0.33 (−0.17 to 0.82)], though there was no difference between subgroups (*P*=0.95; Table 3). The improvement in cardiorespiratory endurance was only observed in studies with a quality score below the median value [SMD_between_ (95 % CI): 0.45 (0.09 to 0.79)], not in those above the median value in quality score [SMD_between_ (95 % CI): 0.22 (−0.07 to 0.51)], but there was no between group difference (*P=*0.33; Table 3). Cardiorespiratory endurance was improved in studies that utilized a maximal exercise testing [SMD_between_ (95 % CI): 0.46 (0.02 to 0.89)], and in those that conducted submaximal exercise testing [SMD_between_ (95 % CI): 0.26 (0.01 to 0.51)].

**Table 3:** Subgroup analyses assessing potential moderating factors for aerobic fitness in controlled studies included in the *meta*-analysis.

Subgroup	Studies		Aerobic Fitness			
	Number	References	SMD (95 % CI)	τ^2^	Within-group *P* value	Between-group *P* value
*Categories of comparator group*						
Usual Care	9	(27, 28, 30, 35–37, 46, 51, 53)	**0.40 (0.13 to 0.68)**	0.10	**0.004**	0.26
Alternative ERS	5	(29, 32, 33, 38, 45)	0.15 (−0.21 to 0.50)		0.41	
*Type of ERS*						
Exercise	8	(28, 30, 33, 35–37, 51, 53)	**0.44 (0.15 to 0.73)**	0.09	**0.003**	0.31
Exercise & Counselling	4	(29, 32, 45, 47)	0.22 (−0.19 to 0.62)		0.30	
Counselling	2	(27, 38)	−0.02 (−0.59 to 0.55)		0.94	
*Duration (weeks)*						
≥ 12	11	(28–30, 32, 37, 38, 45, 47, 51, 53)	**0.31 (0.06 to 0.56)**	0.10	**0.02**	0.95
< 12	3	(33, 35, 36)	0.33 (−0.17 to 0.82)		0.20	
*Methodological Quality*						
≥ 10	8	(27–29, 36, 38, 45, 47, 51)	0.22 (−0.08 to 0.51)	0.10	0.14	0.33
< 10	6	(30, 32, 33, 35, 37, 53)	**0.46 (0.09 to 0.82)**		**0.01**	
*Aerobic Fitness Test*						
Maximal Exercise Testing	5	(29, 32, 37, 38, 51)	**0.46 (0.02 to 0.89)**	0.10	**0.04**	0.44
Submaximal Exercise Testing	9	(27, 28, 30, 33, 35, 36, 45, 47, 53)	**0.26 (0.01 to 0.51)**		**0.04**	

### 3.6 Meta-Analysis of all ERS

**Fig. 3:**
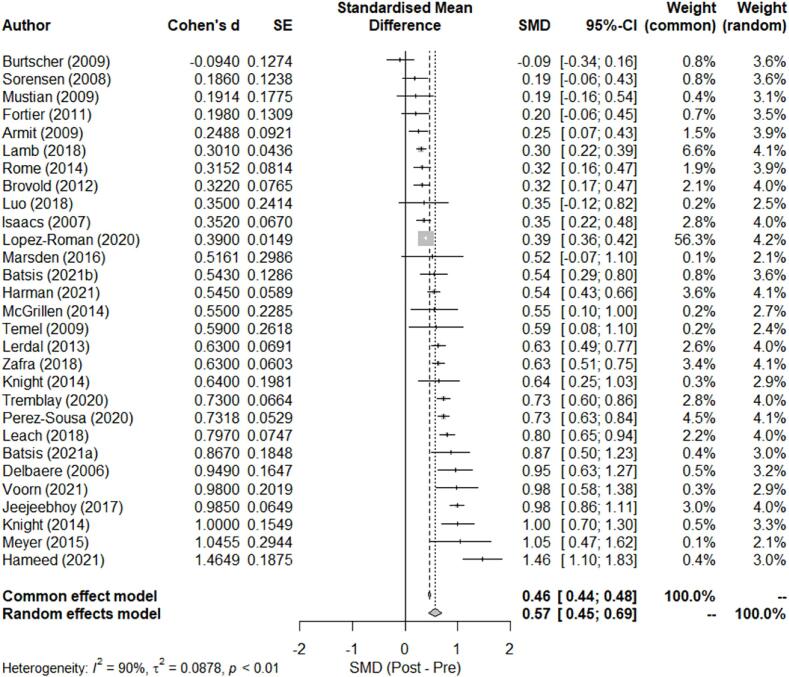
Standardized mean difference (SMD) of the change in cardiorespiratory endurance between Post- and Pre-intervention. Squares represent the SMD for each study. Diamonds represent the pooled SMD across all studies. SE: standard error; CI: confidence interval.

Twenty-nine studies including 5639 participants were included for *meta*-analysis to examine the overall effect of the ERS between pre- and post-intervention. The changes in cardiorespiratory endurance outcomes with the 95 % CIs between pre- and post-intervention for all studies are summarized in a forest plot (Fig. 3). There was a significant improvement in cardiorespiratory endurance following the ERS interventions [SMD_within_ (95 % CI): 0.57 (0.45 to 0.69); Fig. 2] according to the random-effects model, but there was a high level of heterogeneity (I^2^ = 90 %, *P*<0.0001). Cardiorespiratory endurance was improved when the duration of the ERS intervention was shorter than 12-week [8 studies, SMD_within_ (95 % CI): 0.61 (0.38 to 0.84)] or equal to greater than 12-week [21 studies, SMD_within_ (95 %CI): 0.56 (0.41 to 0.70); τ^2^ = 0.09; *P*_between-group_ = 0.71]. In addition, the improvement in cardiorespiratory endurance was observed in studies that used a maximal exercise testing [5 studies, SMD_within_ (95 %CI): 0.28 (0.00 to 0.57)] and in those that utilized a submaximal exercise testing [24 studies, SMD_within_ (95 %CI): 0.62 (0.50 to 0.74); τ^2^ = 0.07; *P*_between-group_ = 0.04].

### 3.7 Risk of bias

Visual inspection of the funnel plot indicated slight asymmetry (Supplemental Digital Content Fig. S4). The Egger’s regression intercepts were 1.23 (*P=*0.27) and 0.39 (*P=*0.052) for SMD_between_ and SMD_within_, respectively. This suggested that publication bias was not present.

## 4 Discussion

The purpose of this study was to review the effectiveness of ERS involving primary care referrals to QEPs on patients’ cardiorespiratory endurance. It was hypothesized that ERS would increase patients’ cardiorespiratory endurance. Consistent with our hypothesis, most studies demonstrated an improvement in patients’ cardiorespiratory endurance levels based on moderate to high quality evidence, as confirmed by the *meta*-analysis results exhibiting a moderate effect size. Such observations support the usefulness of ERS involving QEPs as models to help improve patients’ cardiorespiratory fitness.

Physical activity and cardiorespiratory endurance are inter-related but distinctly different concepts (Bray et al., 2009). Previous reviews of ERS have focused on changes in self-reported physical activity, finding little to no change in patients’ levels of activity before and after the ERS intervention (Pavey et al., 2011, Williams et al., 2007). However, few ERS have been evaluated using objective measures of cardiorespiratory endurance (O’Brien et al., 2021). The discrepancy between the present review and these prior activity focused studies (Pavey et al., 2011, Williams et al., 2007) may be attributed to the measurement of physical activity, whereby self-report measures can be subject to self-report bias or lack sensitivity to detecting change. Specifically, self-report measurements have been demonstrated to be both higher and lower than objective measurements, with minimal consistency (Prince et al., 2008). Conversely, any time spent engaging in light or moderate intensity activity may be replaced by moderate or vigorous activity, resulting in no change in total activity time despite the higher intensity resulting in a training stimulus that improved cardiorespiratory endurance, these measurement considerations may, at least in part, explain the divergent findings between studies. Further differences exist between this review and past reviews (Pavey et al., 2011, Williams et al., 2007) such as the requirement of the person facilitating the exercise being a QEP was not required in prior work (Pavey et al., 2011). Based on our previous scoping review (O’Brien et al., 2021) and surveys of primary care providers (Pellerine et al., 2022), a specific interest in QEP-led interventions was studied. The importance of the educational component of these programs may align with the main tenets of self-determination theory while focusing on the intention-to action stage of the behavioural change process (Garrin, 2014). Self-determination theory relates to how individuals are motivated to engage in daily tasks. This theory focuses on the 3 tenets of autonomy, competence and relatedness (Ryan and Deci, 2000). QEPs may play a role in fostering a sense of self efficacy and self-determination by providing effective support and the learning tools needed by individuals to continue to flourish independently (Garrin, 2014).

An improvement in cardiorespiratory endurance is highly clinically relevant as it has been linked to decreased all-cause mortality and a lower risk of experiencing a cardiovascular event. In older adults, the age-induced graduate loss of muscle mass and cardiovascular function can lead to a longitudinal decline in cardiorespiratory endurance, which, in turn, could limit functional independence and decrease quality of life (Morey et al., 1998). A critical aerobic threshold between 18 and 20 ml/kg/min has been identified, below which there is a significantly reduced probability of independence and a decline in physical function (Cress and Meyer, 2003). In contrast, cardiorespiratory endurance above this threshold may provide an aerobic reserve (i.e., maximal voluntary exceeding the need to perform daily functions) and a “margin of safety” against age-related functional decline (Cress and Meyer, 2003). Accordingly, improving cardiorespiratory endurance through ERS should be considered as a modification strategy to preserve physical function and independence in older adults (Posner et al., 1995). While our review indicated that most current ERS research focuses on middle-aged to older populations, it may be worth considering the potential benefits of introducing ERS as a preventive intervention at a younger age. Early implementation of ERS could increase awareness about the importance of physical activity, enhance intrinsic motivation, and establish healthy exercise habits. This approach would substantially reduce the risk of chronic diseases and improve long-term health outcomes (Reiner et al., 2013). Future studies should explore the effectiveness of ERS in younger populations and determine the longitudinal effects of ERS. Identifying intervention characteristics that allow for the greatest improvement in cardiorespiratory endurance may be useful in the design of future ERS. In the current review, a diverse range of intervention types led to improvement in cardiorespiratory endurance. Fitness measures and exercise types were heterogeneously reported between studies. Based on the subgroup analysis of controlled trials specifically, exercise training was efficacious in improving cardiorespiratory endurance and a greater effect was observed when comparing the intervention to usual care (versus an alternative group). This has also been proposed in past reviews highlighting the absence of differences between control and ERS intervention outcomes when participants who complete an unsupervised bout of training show similar outcomes to those who have supervision (Pavey et al., 2011). Similarly, the *meta*-analysis results of ERS without considering a control/comparator group, more consistently demonstrate an improvement in fitness regardless of length and type of fitness assessment. While lower-quality studies (based on median split) were associated with an improvement in cardiorespiratory endurance in the controlled study *meta*-analysis, it is worth noting that 10/14 is a relatively high median study quality value for evaluating controlled studies. Accordingly, the *lower* quality studies may be more accurately described as *moderate* quality. Nevertheless, this may also be explained by the studies implementing higher intensity training leading to both higher improvements in fitness and higher study dropout that impacts the study quality score. While creating specific guidelines for optimal ERS is difficult due to the heterogeneity observed across successful studies, we suggest an emphasis on reducing dropout, using a maximal exercise test (if possible), and generally an inclusion of cardiorespiratory endurance as an outcome.

In a review of ERS adherence, the main barriers observed to lasting ERS participation was the timing, cost, and location of the intervention (Morgan et al., 2016). Addressing these concerns will increase access to more community members. Ensuring that sessions are not overly long or monotonous may help increase participant enjoyment and consequently retention (Stathi et al., 2004). Reviews of exercise enjoyment have observed participants to prefer group activities but had mixed views on exercise type, with a trend of dislike of gym-based exercises (Morgan et al., 2016). By addressing these factors, future ERS may be able to improve efficacy and reduce participant dropout. Future analyses that consider cost-effective changes in participant cardiorespiratory endurance in addition to physical activity (Anokye et al., 2011) are needed.

## 5 Limitations

While the study is strengthened by considering ERS involving QEPs specifically and conducting a *meta*-analysis on these interventions, it is not without limitations. The main limitations arise from the quality assessment, lack of consistency in individual study reporting, and rigorous requirements for participants, which led to participant attrition. High dropout rates were observed in most of the studies. Dropout has been associated with overcomplicated interventions and difficulty accessing the intervention (Morgan et al., 2016). There was a lack of blinding in most studies, which may have led to biases in assessment. In addition to high dropout, there was a lack of distinct follow up periods in many of the studies. Standardizing follow-up may be helpful in determining the long-term efficacy of interventions. In addition, we found only two studies that performed sex-specific analyses, both of which reported significant improvements in cardiorespiratory endurance in both males and females with no sex difference (Tremblay et al., 2020, Meseguer Zafra et al., 2018). As such, we were not able to perform any *meta*-analyses to examine the impact of sex on cardiorespiratory endurance responses to ERS. Almost half of the studies (*n* = 12) did not state the numbers of male and female participants. Therefore, we recommend that future studies should include sex-specific results and conduct analyses separately for males and females whenever possible. In the present study, only seven studies utilized maximal exercise protocols to assess cardiorespiratory endurance, and only three studies directly assessed maximal oxygen consumption (V̇O_2_max). While submaximal exercise test protocols (e.g., 6MWT) can provide an estimation of cardiorespiratory endurance, they are not as precise as maximal exercise testing (Ross et al., 2016). Due to the inconsistency of cardiorespiratory endurance metrics used across individual studies, we were unable to calculate the specific improvement in maximal oxygen consumption (in ml/kg/min or metabolic equivalents of task) following ERS; instead, we could only determine the overall effect. To better quantify the effects of ERS on cardiorespiratory endurance in varied populations, it would be beneficial to apply direct maximal or symptom-limited exercise testing to more thoroughly evaluate the efficacy of the ERS. Lastly, the description of the referral process was not described in studies, making it challenging to discern whether there are key aspects to the referral specifically that could influence patient uptake (e.g., electronic versus paper, referral uptake, description of referral from provider to patient, etc.).

## 6 Conclusion

ERS linking primary care providers to QEP effectively improve patients’ cardiorespiratory endurance levels based on moderate-high quality evidence. The information gained in this review can be useful for creating and modifying existing ERS in terms of their assessment outlines, cardiorespiratory endurance test, and intervention type. Establishing ERS involving QEPs may be needed to improve patients’ cardiorespiratory endurance on a population level.

Authors’ contributions

MWO conceptualized the study design, developed the search strategy, and inclusion/exclusion criteria. SI, HL, ES, and SR screened identified articles for inclusion, extracted data, and conducted quality assessment of articles. SI, HL, and MWO drafted the manuscript. HL and JG conducted the *meta*-analysis. All authors edited, read, and approved the final manuscript.

## Funding

Not Applicable.

## CRediT authorship contribution statement

**Sophie J.L. Inkpen:** Writing – review & editing, Writing – original draft, Methodology, Formal analysis. **Haoxuan Liu:** Writing – original draft, Methodology, Formal analysis. **Sophie Rayner:** Writing – review & editing, Methodology, Formal analysis. **Ellie Shields:** Writing – review & editing, Methodology, Formal analysis. **Judith Godin:** Writing – review & editing, Methodology, Formal analysis. **Myles W. O’Brien:** Writing – review & editing, Writing – original draft, Supervision, Methodology, Formal analysis, Data curation, Conceptualization.

## Declaration of competing interest

The authors declare that they have no known competing financial interests or personal relationships that could have appeared to influence the work reported in this paper.

## Data Availability

Data will be made available on request.
