# Supplementary material for: Exercise referral schemes increase Patients’ cardiorespiratory Endurance: A systematic review and Meta-Analysis
Source: Prev Med Rep. 2024 Aug 3;45:102844. doi: 10.1016/j.pmedr.2024.102844 (PMC11357876; doi:10.1016/j.pmedr.2024.102844)
Supplement: Supplementary Data 2 [file mmc2.docx]

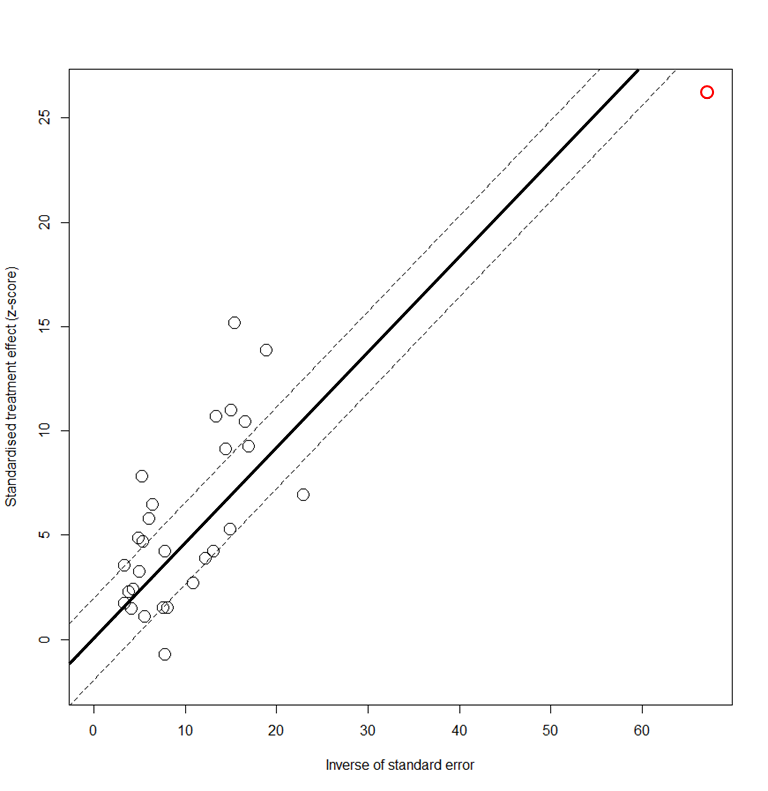
**Supplemental Figure S2: Galbraith plot of standardised effect size (Z-score, Y-axis) by the inverse of the standard errors (Precision, X-axis) for all studies; Dashed lines: 95% confidence interval lines; Red circle: outlier.**
