# Supplementary material for: Exercise referral schemes increase Patients’ cardiorespiratory Endurance: A systematic review and Meta-Analysis
Source: Prev Med Rep. 2024 Aug 3;45:102844. doi: 10.1016/j.pmedr.2024.102844 (PMC11357876; doi:10.1016/j.pmedr.2024.102844)

**Supplemental Figure S3: Galbraith plot of standardised effect size (Z-score, Y-axis) by the inverse of the standard errors (Precision, X-axis) for all studies after removing the outlier (López-Román et al., 2020) identified from Figure S2. Removing this study did not alter the meta-analysis result [standardized mean difference (95% confidence interval): 0.58 (0.45 to 0.70)]. Solid line: regression line; Dashed lines: 95% confidence interval lines.**


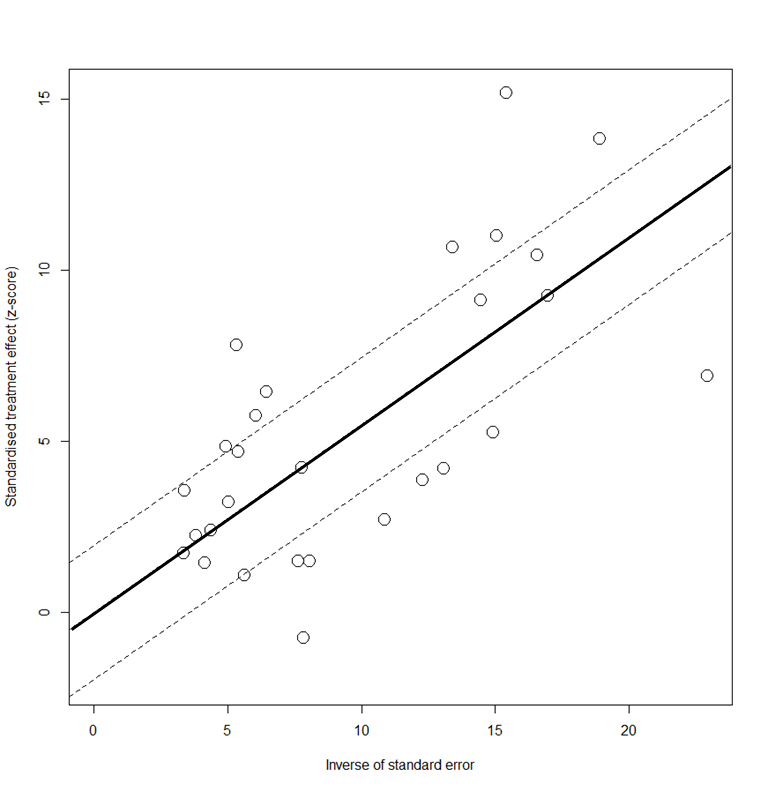

Supplement: Supplementary Data 3 [file mmc3.docx]
