# Supplementary material for: Exercise referral schemes increase Patients’ cardiorespiratory Endurance: A systematic review and Meta-Analysis
Source: Prev Med Rep. 2024 Aug 3;45:102844. doi: 10.1016/j.pmedr.2024.102844 (PMC11357876; doi:10.1016/j.pmedr.2024.102844)
Supplement: Supplementary Data 4 [file mmc4.docx]

**Supplemental Figure S4.** **(A)** Funnel plot of effect size (between-group standardised mean difference) by standard error. **(B)** Funnel plot of effect size (Cohen’s *d*, within-group standardised mean difference) by standard error.
