# Supplementary material for: Exercise referral schemes increase Patients’ cardiorespiratory Endurance: A systematic review and Meta-Analysis
Source: Prev Med Rep. 2024 Aug 3;45:102844. doi: 10.1016/j.pmedr.2024.102844 (PMC11357876; doi:10.1016/j.pmedr.2024.102844)
Supplement: Supplementary Data 5 [file mmc5.docx]

**Table S1.** Search strategy.

| Search | Terms |
| --- | --- |
| Activity Practice Terms | ("Exercise Referral" OR "Activity Referral" OR "Exercise Prescription" OR "Activity Prescription" OR "Exercise Program" OR "Activity Program" OR "Exercise Counselling" OR "Activity Counselling" OR "Exercise Referral Scheme" OR "Exercise Consultation" OR "Activity Consultation") |
| Exercise Terms | "Aerobic" OR "Continuous" OR "Endurance" OR "Resistance" OR "Interval training" OR "Strength training" OR "Physical exercise" OR "Low-intensity" OR "High-intensity" OR "Moderate-intensity" OR "Exercise training" OR "Aerobic training" OR "Yoga" OR "Tai-Chi" OR "Flexibility training" OR "Balance training" OR "Weightlifting" OR "Vigorous exercise" OR "Combined exercise" |
| Provider Terms | "Primary Care" OR "Health Care" OR "Healthcare" OR "Doctor" OR "Physician" OR "Nurse" OR "General Practitioner" OR "Family Medicine" OR "Exercise Professional" OR "Physical Therapist" OR "Physiotherapist" |
| Final Search | Activity Practice Terms AND Exercise Terms AND Provider Terms |
