# Supplementary material for: Exercise referral schemes increase Patients’ cardiorespiratory Endurance: A systematic review and Meta-Analysis
Source: Prev Med Rep. 2024 Aug 3;45:102844. doi: 10.1016/j.pmedr.2024.102844 (PMC11357876; doi:10.1016/j.pmedr.2024.102844)
Supplement: Supplementary Data 6 [file mmc6.docx]

**Table S2: Fitness Intervention Characteristics For All Included Studies**

| **Study** | **Healthcare Provider** | **Intervention Type** | **AF Test** | **Length Of Intervention (Months)** | **Length Of Follow-Up (Months)** | **Session Length (Minutes)** | **Intensity** |
| --- | --- | --- | --- | --- | --- | --- | --- |
| **Armit** | Exercise Scientist | AC | Submaximal Step Test | 12 | 12 | 30 | NR |
| **Sørensen 2008** | General Practitioner | AC And P | Maximal Treadmill Test | 4 | 10 | 60 | > 50% HRR |
| **Romé 2014** | Physician, Nurse, Physiotherapist, Occupational Therapist, Welfare Officer Or Nutritionist. | I1-AC And P  I2--Written P | 6-Min Walk Test | 4 | 12 | 45 | Moderate-Intensity |
| **Lerdal 2013** | Physician | AC And P | 2-Km Walk Test | 3 | 12 | 90 | Moderate |
| **Burtscher 2009** | Family Physician | I1-AC And P I2-AC Only | Maximal Cycle Ergometer Test | 12 |  | 30 | Moderate-Intensity (Blood Lactate Levels Of 2 To 3 Mmol/L) |
| **Isaacs 2007** | General Practitioner | I1 = Supervised Training ; I2 = Activity P I3 = AC And P | Submaximal Cycle Ergometer Test. | 3.5 | 12 | 45 | Individual To Participants |
| **Leach 2018** | Oncology Clinicians Or Staff | EP | 6-MinWalk Test | 3 | NR | 60 | RPE 11-14 |
| **Hameed 2021** | Physician | EP | 2-Min Step Test | 1.25 | 0.5 | 45 | Maintain Spo2 > 88% During Exercise |
| **Mustian 2009** | Physician | EP | 6-MinWalk Test | 1 | 3 | NR | 60-70% Of HRR |
| **Marsden 2016** | Clinician | Home And Community Based EP | Maximal Cycle Ergometer Test | 3 | NR | 30 | Moderate Intensity |
| **Fortier 2011** | Physician | I1 = EP ; I2 = Exercise Counselling | Maximal Treadmill Test | 6 | NR | Supervised | Aerobic |
| **Luo 2023** | Oncologist | Supervised EP | 400-m Walk Test | 3 | 3 | 60 | Resistance Exercise: 2-4 Sets At 6-12 RM Aerobic: 60-85% Hrmax |
| **McGrillen 2014** | Physiotherapist, Palliative Care Consultant, Occupational Therapist, General Practitioner. | Exercise Counselling And P | 6-Min Walk Test | 2.5 | NR | 60 | RPE 12-14 |
| **Lamb 2018** | General Practitioners And Clinicians | AC, EP | 6-MinWalk Test | 4 | 1.5 | 60 | Moderate To Moderate To Hard Level |
| **López-Román 2020** | General Practitioners And Nurses | Activity P And Supervised Training | 2-Km Walking Test | 3.5 | NR | 60 | The Modified Borg Dyspnoea Scale [19] Was Used |
| **Temel 2009** | Thoracic Oncology Clinicians | EP And Supervised Training | 6-Min Walk Test | 2 | 105 | Supervised | 70-85% Hrmax Or RPE 13. |
| **Harman 2021** | Physician | EP | Submaximal Treadmill Test (UNCCRI) | 3 | NR | 60 | 30-60% HHR Based On Karvonen Formula |
| **Jeejeebhoy 2017** | Physician | Dietary And EP | Submaximal Treadmill Test (Ebbeling) | 12 | NR | 45 | 50 To 75% Hrmax |
| **Brovold 2012** | Physician | AC And P (More In Depth P For The IT Group). | 6-MinWalk Test | 3 | 3 | 45 | Low Intensity Resistance Training |
| **Knight 2014** | Primary Care Practitioners | AC And P | Submaximal STEP Test | 3 | 9 | NR | Moderate Endurance |
| **Batsis 2021a** | Physicians | Dietary Intervention, AC, And Exercise Training (Home And On-Site) | 6-MinWalk Test | 12 | NR | NR | Moderate-Vigorous |
| **Tremblay 2020** | Family Physician | EP And Supervised Training | Submaximal Treadmill Test (Ebbeling) | 12 | NR | 35 | First 3 Months: 50% Maximal Heart Rate  After 3 Months: 65-75% Of Maximal Heart Rate |
| **Batsis 2021b** | Physician | AC And Training | 6-Min Walk Test | 6.5 | NR | 75 | Moderate Intensity |
| **Meyer 2015** | Pulmonary Specialist | Training Only | Maximal Cycle Ergometer Test | 12 | NR | 60 | > 60% Max HR |
| **Voorn 2021** | Rehabilitation Physicians | EP | Submaximal Cycle test | 4.5 | NR | 20 | Low Intensity: <RPE12  High Intensity: >RPE12. |
| **Delbaere 2006** | General Practitioner Or Physiotherapist | Individual Training Sessions Over The Trial Duration | 6-MinWalk Test | 4 | NR | 30 | RPE 12 To 14 |
| **Zafra 2018** | "Primary Care" Not Specified | Exercise Training | 1-mile walk test | 2.5 | NR | 60 | 60-80% HR Max |
| **Perez-Sousa 2020** | General Practitioners | Exercise Training | 6-Min Walk Test | 12 | NR | 50 | Moderate |
| **Knight 2014** | Physician | EX = Personalized Home-Based Daily Eps  SB = AC And Personalized P CC = Physical Activity Prescription | Submaximal STEP Test | 3 | NR | 10 | 65-85% Of Max HR |
