# Supplementary material for: Exercise referral schemes increase Patients’ cardiorespiratory Endurance: A systematic review and Meta-Analysis
Source: Prev Med Rep. 2024 Aug 3;45:102844. doi: 10.1016/j.pmedr.2024.102844 (PMC11357876; doi:10.1016/j.pmedr.2024.102844)
Supplement: Supplementary Data 7 [file mmc7.docx]

Table S3: NIH Quality assessment checklist for studies with a control group (n=15)

| With Control Group | | | | | | | | | | | | | | | |
| --- | --- | --- | --- | --- | --- | --- | --- | --- | --- | --- | --- | --- | --- | --- | --- |
|  | Sørensen 2008 | Romé 2014 | Burtscher 2009 | Isaacs 2007 | Hameed 2021 | Mustian 2009 | Marsden 2016 | Fortier 2011 | Lamb 2018 | Brovold 2012 | Meyer 2015 | Delbaere 2006 | Perez-Sousa 2020 | Knight 2014 | Armit 2009 |
| 1. Was the study described as randomized, a randomized trial, a randomized clinical trial, or an RCT? | 1 | 1 | 1 | 1 | 0 | 1 | 0 | 1 | 1 | 1 | 1 | 0 | 0 | 1 | 1 |
| 2. Was the method of randomization adequate (i.e., use of randomly generated assignment)? | 1 | 1 | 1 | 1 | 0 | 1 | 0 | 1 | 1 | 1 | 0 | 0 | 0 | 1 | 1 |
| 3. Was the treatment allocation concealed (so that assignments could not be predicted)? | 1 | 1 | 0 | 1 | 0 | 1 | 0 | 1 | 1 | 1 | 0 | 0 | 0 | 1 | 1 |
| 4. Were study participants and providers blinded to treatment group assignment? | 0 | 0 | 0 | 0 | 0 | 1 | 0 | 0 | 0 | 0 | 0 | 0 | 0 | 0 | 1 |
| 5. Were the people assessing the outcomes blinded to the participants' group assignments? | 0 | 0 | 0 | 0 | 0 | 1 | 1 | 1 | 1 | 1 | 1 | 0 | 0 | 0 | 0 |
| 6. Were the groups similar at baseline on important characteristics that could affect outcomes (e.g., demographics, risk factors, co-morbid conditions)? | 1 | 1 | 1 | 1 | 1 | 1 | 1 | 1 | 1 | 1 | 1 | 1 | 1 | 1 | 1 |
| 7. Was the overall drop-out rate from the study at endpoint 20% or lower of the number allocated to treatment? | 0 | 0 | 0 | 0 | 0 | 1 | 1 | 1 | 1 | 0 | 1 | 0 | 1 | 1 | 1 |
| 8. Was the differential drop-out rate (between treatment groups) at endpoint 15 percentage points or lower? | 1 | 1 | 0 | 0 | 0 | 1 | 1 | 1 | 1 | 1 | 1 | 0 | 1 | 1 | 1 |
| 9. Was there high adherence to the intervention protocols for each treatment group? | 1 | 0 | 0 | 0 | 0 | 1 | 1 | 1 | 0 | 1 | 1 | 0 | 0 | 0 | 1 |
| 10. Were other interventions avoided or similar in the groups (e.g., similar background treatments)? | 1 | 1 | 1 | 1 | 1 | 1 | 1 | 1 | 1 | 1 | 1 | 1 | 1 | 1 | 1 |
| 11. Were outcomes assessed using valid and reliable measures, implemented consistently across all study participants? | 1 | 1 | 1 | 1 | 1 | 1 | 1 | 1 | 1 | 1 | 1 | 1 | 1 | 1 | 1 |
| 12. Did the authors report that the sample size was sufficiently large to be able to detect a difference in the main outcome between groups with at least 80% power? | 1 | 0 | 0 | 0 | 0 | 0 | 0 | 0 | 1 | 0 | 1 | 1 | 1 | 0 | 1 |
| 13. Were outcomes reported or subgroups analyzed prespecified (i.e., identified before analyses were conducted)? | 1 | 1 | 1 | 1 | 1 | 1 | 1 | 1 | 1 | 1 | 1 | 1 | 1 | 1 | 0 |
| 14. Were all randomized participants analyzed in the group to which they were originally assigned, i.e., did they use an intention-to-treat analysis? | 1 | 1 | 1 | 0 | 0 | 1 | 1 | 1 | 1 | 1 | 0 | 0 | 0 | 1 | 1 |
