# Supplementary material for: Exercise referral schemes increase Patients’ cardiorespiratory Endurance: A systematic review and Meta-Analysis
Source: Prev Med Rep. 2024 Aug 3;45:102844. doi: 10.1016/j.pmedr.2024.102844 (PMC11357876; doi:10.1016/j.pmedr.2024.102844)
Supplement: Supplementary Data 8 [file mmc8.docx]

Table S4: NIH Quality assessment checklist for studies without a control group (n=14)

| No Control Group | | | | | | | | | | | | | | |
| --- | --- | --- | --- | --- | --- | --- | --- | --- | --- | --- | --- | --- | --- | --- |
|  | Lerdal 2013 | Leach 2018 | Luo 2023 | McGrillen 2014 | López-Román 2020 | Temel 2009 | Harman 2021 | Jeejeebhoy 2017 | Knight 2014 | Batsis 2021 | Tremblay 2020 | Batsis 2021 | Voorn 2021 | Zafra 2018 |
| 1. Was the study question or objective clearly stated? | 1 | 1 | 1 | 1 | 1 | 1 | 1 | 1 | 1 | 1 | 1 | 1 | 1 | 1 |
| 2. Were eligibility/selection criteria for the study population prespecified and clearly described? | 1 | 1 | 1 | 1 | 1 | 1 | 1 | 1 | 1 | 1 | 1 | 1 | 1 | 1 |
| 3. Were the participants in the study representative of those who would be eligible for the test/service/intervention in the general or clinical population of interest? | 0 | 1 | 1 | 1 | 1 | 0 | 1 | 1 | 1 | 1 | 1 | 1 | 1 | 1 |
| 4. Were all eligible participants that met the prespecified entry criteria enrolled? | 0 | 1 | 1 | 0 | 1 | 1 | 1 | 1 | 1 | 0 | 1 | 1 | 1 | 0 |
| 5. Was the sample size sufficiently large to provide confidence in the findings? | 1 | 0 | 0 | 0 | 1 | 0 | 0 | 1 | 0 | 0 | 1 | 1 | 0 | 0 |
| 6. Was the test/service/intervention clearly described and delivered consistently across the study population? | 0 | 1 | 1 | 1 | 1 | 1 | 0 | 1 | 0 | 1 | 1 | 1 | 1 | 1 |
| 7. Were the outcome measures prespecified, clearly defined, valid, reliable, and assessed consistently across all study participants? | 0 | 1 | 1 | 1 | 1 | 1 | 1 | 1 | 1 | 1 | 1 | 1 | 1 | 1 |
| 8. Were the people assessing the outcomes blinded to the participants' exposures/interventions? | 0 | 0 | 0 | 0 | 0 | 0 | 0 | 0 | 0 | 0 | 0 | 0 | 0 | 0 |
| 9. Was the loss to follow-up after baseline 20% or less? Were those lost to follow-up accounted for in the analysis? | 0 | 0 | 0 | 0 | 1 | 0 | 1 | 1 | 1 | 1 | 0 | 1 | 1 | 1 |
| 10. Did the statistical methods examine changes in outcome measures from before to after the intervention? Were statistical tests done that provided p values for the pre-to-post changes? | 1 | 1 | 1 | 1 | 1 | 1 | 1 | 1 | 0 | 1 | 1 | 1 | 1 | 1 |
| 11. Were outcome measures of interest taken multiple times before the intervention and multiple times after the intervention (i.e., did they use an interrupted time-series design)? | 0 | 0 | 0 | 0 | 0 | 0 | 0 | 0 | 0 | 0 | 0 | 1 | 0 | 0 |
| 12. If the intervention was conducted at a group level (e.g., a whole hospital, a community, etc.) did the statistical analysis take into account the use of individual-level data to determine effects at the group level? | 0 | 0 | 0 | 0 | 0 | 0 | 0 | 0 | 0 | 0 | 0 | 0 | 0 | 0 |
